# Effect of conventional grain-fed and grass-fed feeding systems on fecal microbiota and shiga toxin-producing *Escherichia coli* in beef cattle

**DOI:** 10.1186/s12866-025-04073-6

**Published:** 2025-06-06

**Authors:** Sudipta Talukder, Frederick Yang, Sarah Klopatek, James Oltjen, Xiang Yang

**Affiliations:** Department of Animal Science, University of California-Davis, 2237 Meyer Hall, Davis, CA 95616 USA

**Keywords:** STEC, Microbiota, Grain-fed, Grass-fed, Beef

## Abstract

**Background:**

Shiga toxin-producing *Escherichia coli* (STEC) remains a significant public health concern in beef production, regardless of feeding systems. While consumer interest in grass-fed beef has increased due to climate change concerns and social media trends, the safety implications of different feeding practices on STEC prevalence and populations are not fully understood. Therefore, the objectives of this study were to evaluate the effects of grain-fed and grass-fed feeding systems on STEC prevalence, population, and its interaction with the fecal microbiota in beef cattle. Post-weaning steers were assigned to four feeding systems: Conventional grain-fed (CON, *n* = 21), 20-month grass-fed (20GF, *n* = 18), 25-month grass-fed (25GF, *n* = 16), and 45-day grain-fed after 20-month grass-fed (GR45, *n* = 13). Rectal fecal samples were collected at 14 months of age as baseline and pre-harvest for STEC enumeration, prevalence, and microbial analysis. The microbial DNA was extracted and sequenced for 16 S rRNA gene for microbiota analysis.

**Results:**

Data demonstrated that cattle in grain-fed feeding system had a higher (*P* < 0.05) fecal STEC population than the grass-fed feeding system. However, the fecal prevalence of STEC was lower (*P* < 0.05) only in the GR45 compared to the grass-fed groups, while the CON group did not differ (*P* > 0.05) in STEC prevalence. In terms of STEC population, GR45 was more similar to the grain-fed group. Alpha diversity was greater (*P* < 0.05) in CON, followed by 25GF, with GR45 being the only system where alpha diversity decreased (*P* < 0.05) from baseline to harvest. Beta diversity showed a notable difference (*R* = 0.913, *P* = 0.001) in fecal microbial composition between CON and GR45. Firmicutes and Bacteroidetes were the dominant phyla across all feeding systems. At harvest, GR45 had the highest (*P* < 0.0001) Firmicutes abundance, followed by 20GF, while the lowest levels were observed in 25GF and CON. Among bacterial families, *Peptostreptococcaceae* was more abundant in grass-fed groups, whereas *Ruminococcaceae* was more prevalent in the grain-fed CON group. Microbiota associated with lower STEC prevalence, such as Bacteroidetes, were more abundant in STEC-negative samples.

**Conclusions:**

These findings suggest that feeding systems influence both STEC levels and gut microbial diversity, offering insights into managing microbiota to enhance food safety in beef production. Nonetheless, the results should be interpreted in the context of the study’s limited sample size and the inherent variability associated with intermittent STEC shedding and microbiota composition.

## Background

Cattle serve as a reservoir for a wide array of microorganisms, some of which are pathogenic to humans [1]. When these pathogens enter the food production chain, they jeopardize the safety of beef products, creating significant risks to human health and causing detrimental impacts on both consumers and producers. Shiga toxin-producing *Escherichia coli* (STEC), such as the deadly *Escherichia coli* O157:H7, are prevalent foodborne pathogens present in the gastrointestinal tract and fecal matter of cattle [2]. An annual toll of over 265,000 human illnesses, 3,600 hospitalizations, and 30 fatalities due to STEC infection was estimated in the United States (US) [3]. Despite underreporting of illnesses linked to STEC, recent trends show that STEC infection rates have remained relatively stable in the US. According to the latest data from 2022, the incidence of laboratory-diagnosed, domestically-acquired STEC infections was 4.6 per 100,000 population, which is unchanged from the 2016–2018 baseline [4]. However, it’s important to note that STEC infections in humans are associated with various sources, including beef cattle, fresh produce, and other food products.

Various factors, such as diet, age, and external stressors, have been demonstrated to impact the microbiota and loads of pathogens in cattle’s gastrointestinal tract [5–7]. A prolonged discussion has persisted regarding the utilization of grain and grass diets in cattle production, covering concerns ranging from sustainability and palatability to issues related to food safety [8, 9]. While grass-fed beef currently accounts for 4–5% of the US beef market, there has been a steady rise in its consumption over the last decade [10]. The grass-fed beef market is projected to grow by nearly $3.7 billion between 2023 and 2028, with a compound annual growth rate (CAGR) of 5.5% [11]. Reports indicate that consumers’ inclination toward grass-fed beef is shaped by various factors, such as environmental sustainability, animal welfare, and total fat profile [12]. Previous studies comparing the nutritional disparities between grass-fed and grain-fed beef in the U.S. indicated that while grass-fed beef yields leaner meat with lower saturated fatty acids, it also contains a lower percentage of monounsaturated and polyunsaturated fatty acids, which are considered beneficial for human health [13, 14]. Comparable nutritional trade-offs were observed between grass-fed and grain-fed US beef in terms of antioxidants and mineral contents. Regarding quality, certain studies have noted that US grass-fed beef exhibits less tenderness [15], a low marbling score [16], and an off-flavor compared to grain-fed beef [13]. Although the nutritional aspect may be compromised, it is crucial to comprehend the impact of grass-fed feeding system on meat safety to provide insights into the ongoing debate.

Concerns about food safety have escalated over time, as certain connections have been made between grain-fed feeding systems and elevated levels of gastrointestinal pathogenic *E. coli* and acid-resistant *E. coli* [17]. Concurrently, others have observed that forage diets demonstrate an increased fecal STEC population relative to grain diets [18, 19]. These discrepancies in the literature may stem from differences in ruminal fermentation patterns, gut pH, volatile fatty acid production, or microbial community structure, which can vary depending on diet composition, forage type, and animal age. Such variation highlights the complexity of the relationship between feeding systems and STEC shedding and underscores the need for further investigation. Previous research studying the impact of feeding systems on pathogen load has generally focused on the impact of individual aspects that differ between production systems, such as types of grain and forage, moisture content, and animal density [20, 21]. However, there is relatively limited exploration in evaluating the collective impact of these production systems, establishing a baseline for comparison between systems as they operate in real-world conditions. The persistent risk of environmental contamination from cattle-borne pathogens, coupled with the increasing consumer interest in alternative beef production systems, underscores the importance of approaches investigating the differences in pathogen load between production systems, with a particular focus on studying the fecal microbiota as an indicator of overall herd health and food safety risk [9]. In addition to pathogen load, previous research has shown that feeding patterns influence the diversity and composition of the bovine gut microbiota, which plays a critical role in pathogen colonization resistance and overall gut health [6, 7]. Studies have reported that grass-fed cattle generally exhibit higher microbial diversity and distinct community structures compared to grain-fed cattle, often with increased abundance of fiber-degrading or SCFA-producing genera such as *Planctomycetes*, *Lachnospira*, and *Sutterella* [22, 23]. However, the association between microbiota composition and STEC shedding remains inconsistent, with some studies linking reduced diversity to increased STEC prevalence, while others find no clear relationship [24, 25]. Moreover, many of these studies have been conducted under experimental or challenge conditions, rather than in commercial-like environments. As such, integrated evaluations of fecal microbiota and STEC dynamics across diverse, realistic feeding systems remain limited. Furthermore, another animal feeding system that has gained popularity involves transitioning cattle from a grass-fed diet to a grain-finished diet for a short period before harvest. This approach aims to balance the perceived health and environmental benefits of grass feeding with the enhanced marbling and flavor characteristics associated with grain finishing. While this feeding system is distinctive, there is currently a lack of comprehensive data to understand its impact on microbial communities and the occurrence of pathogenic bacteria compared to other feeding systems. To address this gap, the current study evaluated a 45-day grain-finishing period, reflecting a common commercial practice in California that seeks to improve carcass quality without fully altering the microbiota developed during long-term grass feeding. While longer finishing durations (e.g., 60 or 90 days) may induce greater microbiome shifts, this timeframe allowed us to examine the realistic, industry-relevant effects of short-term grain exposure.

Therefore, the objectives of this study were to (1) evaluate the impact of grain-fed (cattle that has been raised in a feedyard for more than 60 days before being processed) and grass-fed (cattle that consumed grass throughout its entire life cycle as per USDA definition [26]) feeding systems currently being followed in California on the population and prevalence of STEC and acid-resistant STEC in the feces of beef cattle; (2) assess the effects of these feeding systems on the fecal microbial communities, and (3) determine whether there is an association between fecal microbiota and STEC prevalence and population.

## Methods

### Study design

The study was performed according to protocols approved by the Institutional Animal Care and Use Committee at the University of California, Davis (UCD; protocol #20560). The steers used in this study were owned by the UCD and managed under IACUC-UCD-approved research and husbandry protocols. Details of the study design were previously described by Klopatek et al. [16]. In brief, Angus and Angus-Hereford cross beef steers owned by the UCD were fence-lined weaned in June 2018, blocked by weight, and randomly assigned to one of the four feeding systems: (1) Conventional grain-fed (CON), (2) 20 months grass-fed (20GF), (3) 25 months grass-fed (25GF), and (4) 45 days grain-fed after 20 months grass-fed (GR45). The number of steers per treatment was adjusted since some steers developed pinkeye infections during the study and were treated with antibiotics per IACUC protocols. Since as per natural program agreements, grass-fed cattle must be free from antibiotics and hormones for processing at a natural plant, the treated steers belonging to the grass-fed groups were assigned to GR45. Consequently, the final cattle counts were: 21 in CON, 18 in 20GF, 16 in 25GF, and 13 in GR45. All steers were stocked on irrigated summer pastures in Maxwell, CA, between June and November 2018. In late November, steers of different feeding systems were transported to different locations. The CON steers were moved to the UCD feedlot (Davis, CA), provided a conventional corn-based feedlot diet for 120 days, and harvested. The 25GF, 20GF, and GR45 steers were moved to the Sierra Foothill Research and Extension Center, Browns Valley, CA, grazed on winter-spring pasture, consisting of grasses (e.g., *Bromus* and *Avena* spp.) and forbs (e.g., *Erodium*, *Medicago*, and *Trifolium* spp.). At 20 months of age following the end of the winter-spring grazing season, the 20GF steers were harvested in June 2019. The GR45 cattle were moved to the UCD feedlot (Davis, CA) and finished on a high-energy corn diet for 45 days before harvest. The composition of feedyard diets for the CON and GR45 steers can be found in Klopatek et al. [16]. The primary difference between the two diets was the duration and backgrounding of the steers: CON steers received a conventional high-energy corn-based diet for 120 days, while GR45 steers transitioned from a long-term forage-based system and were finished on a similar corn-based diet for 45 days. No direct-fed microbials (DFMs) were used in either diet. The 25GF cattle were moved to irrigated pastureland at the UCD (Davis, CA), which consisted of a mixture of perennial grasses and clover. The cattle were then harvested at 25 months of age. Monensin was included in the feed of CON and GR45 steers to replicate commercial feeding systems while no antibiotics or growth hormones were given to grass-fed cattle. Rectal grab fecal samples were obtained individually from all beef cattle at baseline (14 months of age as a baseline prior to the assignment to feeding systems) and one week before harvesting. For each animal, about 50 g of rectal feces were collected each time, deposited into separate sterile sampling bags, immediately stored in coolers with ice, and transported back to the lab at the UCD within 2 h for further processing.

### Isolation and enumeration of STEC and acid-resistant STEC

For each sample, 10 g feces were weighed and mixed in 90 ml modified tryptic soy broth (TSB; Difco, Becton Dickinson Microbiology Systems, Sparks, MD) with phosphate buffer, and homogenized using a Masticator blender (Neutec Group, Farmingdale, NY). An additional 10 g of feces were weighed, placed in a 50mL Eppendorf conical tube (Eppendorf, Hamburg, Germany), and immediately stored in a -80ºC freezer for subsequent fecal microbiota analysis. To enumerate the general STEC population, homogenized fecal samples were serially diluted with buffered peptone water (BPW), plated on CHROMagar STEC plates (CHROMagar Microbiology, Paris, France), and incubated at 35ºC for 24 h. To assess general STEC prevalence, the homogenized fecal samples were streaked onto CHROMagar STEC plates after enrichment at 42ºC for 6 h. Presumptive STEC isolates were arbitrarily selected from CHROMagar STEC plates (2–3 colonies/sample) for PCR confirmation via amplification of genes *stx1*, *stx2*, and *eaeA* on a Bio-Rad T100 Thermal Cycler (Bio-Rad, Hercules, CA) [27]. Isolates were considered STEC-positive if they carried the *eaeA* gene along with either *stx1* or *stx2*. The prevalence of acid-resistant STEC was assessed using a method slightly modified from that described by Kim et al. [28]. Briefly, five presumptive STEC colonies isolated from each sample were arbitrarily selected and inoculated into TSB and incubated at 35ºC for 24 h to ensure active growth and consistent physiological state prior to acid challenge. Then, 50µL of the STEC inoculum was challenged in acidified TSB without dextrose (adjusted to pH of 3.50 with HCl) at 35ºC and streaked onto tryptic soy agar (TSA) after 1 h and after 6 h. The TSA plates were incubated at 35ºC for 24 h to determine the prevalence of the acid-resistant STEC population. The presumptive STEC isolates were confirmed by the same PCR procedures mentioned above, targeting the same set of genes.

### DNA extraction, library preparation, and sequencing

Frozen fecal samples were thawed to 4ºC in a refrigerator overnight. The DNA extraction, library preparation, and sequencing proceeded using the methodology of Jinno et al. [29]. The DNA was extracted from fecal samples using a Quick-DNA Fecal/Soil Microbe Kit (Zymo Research, Irvine, CA, USA) following manufacturer instructions. The extracted DNA was amplified in triplicate via PCR targeting the V4 region of the 16S rRNA gene on a Bio-Rad T100 Thermal Cycler (Bio-Rad, Hercules, CA). Primers used were 515F (5’-XXXXXXXXGTGTGCCAGCMGCCGCGGTAA-3’) with 8 bp barcode(X) and Illumina adapter (GT) modifications and 806R (5’- GGACTACHVGGGTWTCTAAT-3’). Individual samples were given unique 8 bp forward barcode primers (Thermo Fisher Scientific Inc., Waltham, MA). Triplicate PCR products were pooled per sample and confirmed on a 2% agarose gel for successful amplification. The concentration of purity of each sample was quantified using Qubit 4 (Invitrogen, Carlsbad, CA), and Nanodrop 2000 (Thermo Fisher Scientific Inc., Waltham, MA) was used to determine 260/280 and 260/230 absorbance ratios. All PCR samples were pooled together and purified using a QIAquick PCR Purification Kit (QIAGEN, Hilden, Germany). The purified product was sent to the UCD Genome Center DNA Technologies Core for sequencing on the Illumina MiSeq platform (250 bp paired-end, Illumina, Inc., San Diego, CA, USA).

### Bioinformatics analysis

Raw sequences were demultiplexed using sabre (https://github.com/najoshi/sabre) software tool while removing the barcode sequences. Sequences were imported into the Quantitative Insights into Microbial Ecology version 2020.2 (QIIME 2) platform for analysis [30]. The DADA2 plugin was used for sequence quality control, and trimming of the 8 bp barcode primer sequences [31]. Amplicon sequence variants (ASVs) were aligned via mafft [32]. The feature-classifier plugin classify-sklearn naïve Bayes taxonomy classifier [33] was used to assign taxonomy to ASVs, using the SILVA rRNA database release 132 [34] as a reference for training classifiers.

### Statistical analyses

The data for feeding system effects and age effects on beef cattle fecal STEC population and prevalence were analyzed following a completely randomized design with fixed effects of feeding system and age and their corresponding interaction. Feeding system effects were defined as the difference in STEC population and prevalence of different feeding systems at harvest. Age effects were defined as the differences in STEC population and prevalence from fecal samples taken at the post-weaning baseline and one week prior to harvest. Both feeding system effects and age effects on STEC population were analyzed using ANOVA with Tukey’s HSD test for means separation. Feeding system effects and age effects on general STEC prevalence and acid-resistant STEC prevalence were analyzed using Fisher’s exact test.

Alpha diversity of the fecal microbiota was assessed using Chao1 and Shannon indices. Beta diversity was assessed using principal component analysis (PCA). Relative abundances were calculated using the tidyverse and dplyr packages after the read counts were normalized using the cumulative sum scaling normalization in metagenomeSeq package [35]. Two-way ANOVA was used to compare the relative abundances of bacteria at phylum and family levels among different feeding systems. The multivariate homogeneity of group dispersions (number of permutations = 999) and microbial compositional difference between the levels of treatments (feeding systems and time points) were tested using betadisper and adonis functions in vegan package [36], respectively. Correlation between the beta diversity (calculated using the Bray-Curtis dissimilarity) and STEC population and prevalence was conducted by using the Spearman correlation method in Mantel test and PERMANOVA, respectively. All visualizations were built through the ggplot2 package [37]. All statistical analyses and data visualizations were conducted in RStudio 2022.02.3 with confidence level ≥ 95% [38].

## Results

### Enumeration and prevalence of STEC and acid-resistant STEC

The enumerated population and prevalence of STEC and acid-resistant STEC are summarized in Tables 1, 2 and 3. At baseline, there was no difference (*P* > 0.05) in STEC population among feeding systems (Table 1). From baseline to harvest, the fecal STEC population in CON steers increased (*P* < 0.05) by 2.62 log10 CFU/g, while in feces collected from 20GF and 25GF steers STEC population decreased (*P* < 0.05) by 1.69, and 2.04 log10 CFU/g, respectively. At harvest, the fecal STEC population of two grass-fed feeding systems (20GF and 25GF) was found to be lower (*P* < 0.05) than the two grain-fed feeding systems (CON and GR45). For fecal STEC prevalence, there was no difference (*P* > 0.05) between the feeding systems at baseline (Table 2). However, only the GR45 had a decreased (*P* < 0.05) fecal STEC prevalence from baseline (100%) to harvest (46%). Furthermore, at harvest, the fecal STEC prevalence of the GR45 feeding system was lower (*P* < 0.05) than the prevalence of two grass-fed feeding systems (20GF and 25GF). At both baseline and harvest, there was no difference (*P* > 0.05) observed in the prevalence of acid-resistant STEC in the feces among the feeding systems (Table 3). However, there was a decrease (*P* < 0.05) in the prevalence of acid-resistant STEC in the feces of 20GF and GR45 steers from baseline to harvest.

**Table 1:** Shiga toxin-producing Escherichia coli (STEC) populations in fecal samples of beef cattle raised under different feeding systems, assessed at both baseline and harvest time points

	STEC population (log10 CFU/g)	
Treatment^1^	Baseline^2^	Harvest
CON (*n* = 21)	2.78 (0.7)^x^	5.4 (0.7)^a, y^
20GF (*n* = 18)	3.48 (0.7)^x^	1.79 (0.5)^b, y^
25GF (*n* = 16)	3.1 (0.7)^x^	1.06 (0.4)^b, y^
GR45 (*n* = 13)	3.78 (0.7)^x^	4.12 (0.7)^a, x^

^1^ Feeding system treatments: Conventional grain-fed (CON), 20 months grass-fed (20GF), 25 months grass-fed (25GF), and 20 months grass-fed and 45 days grain-fed (GR45)

^2^ No significant difference in STEC population was found between the treatments at baseline

^ab^ Least square means with different superscripts in the column ‘harvest’ are significantly different (*P* < 0.05)

^xy^ Least square means with different superscripts between baseline and harvest in the same row are significantly different (*P* < 0.05)

**Table 2:** Prevalence of STEC detected in fecal samples from beef cattle across various feeding systems at baseline and harvest

	General STEC prevalence (%)	
Treatment^1^	Baseline^2^	Harvest
CON (*n* = 21)	100^x^	82^ab, x^
20GF (*n* = 18)	100^x^	100^a, x^
25GF (*n* = 16)	100^x^	94^a, x^
GR45 (*n* = 13)	100^x^	46^b, y^

^1^ Feeding system treatments: Conventional grain-fed (CON), 20 months grass-fed (20GF), 25 months grass-fed (25GF), and 20 months grass-fed and 45 days grain-fed (GR45)

^2^ No significant difference in STEC prevalence was found between the treatments at baseline

^ab^ Values with different superscripts in the column ‘harvest’ are significantly different (*P* < 0.05)

^xy^ Values with different superscripts between baseline and harvest in the same row are significantly different (*P* < 0.05)

**Table 3:** Acid-resistant STEC prevalence in fecal samples collected from beef cattle subjected to different feeding systems, evaluated at two time points: baseline and harvest

	Acid-resistant^4^ STEC prevalence (%)	
Treatment^1^	Baseline^2^	Harvest^3^
CON (*n* = 21)	18.18^x^	0^x^
20GF (*n* = 18)	41.18^x^	5.88^y^
25GF (*n* = 16)	31.25^x^	12.5^x^
GR45 (*n* = 13)	46.15^x^	0^y^

^1^ Feeding system treatments: Conventional grain-fed (CON), 20 months grass-fed (20GF), 25 months grass-fed (25GF), and 20 months grass-fed and 45 days grain-fed (GR45)

^2^ No significant difference in acid-resistant STEC prevalence was found between the treatments at baseline

^3^ No significant difference in acid-resistant STEC prevalence was found between the treatments at harvest

^4^ Acid-resistant STEC are those that survived an acid challenge (pH 3.5, tryptic soy broth without dextrose, 6 h)

^xy^ Values with different superscripts between baseline and harvest in the same row are significantly different (*P* < 0.05)

### Sequencing metrics

A total of 2,687,477 reads were obtained from all fecal samples with an average of 20,360 reads per sample after removing poor-quality reads. The ASVs were filtered to remove features with low abundance and seen in less than 10% of the samples. A final total of 2,219,692 reads from 878 ASVs were retained from the initial 10,419 features detected, representing 82% of total reads.

### Diversity of the fecal Microbiome

**Fig. 1:**
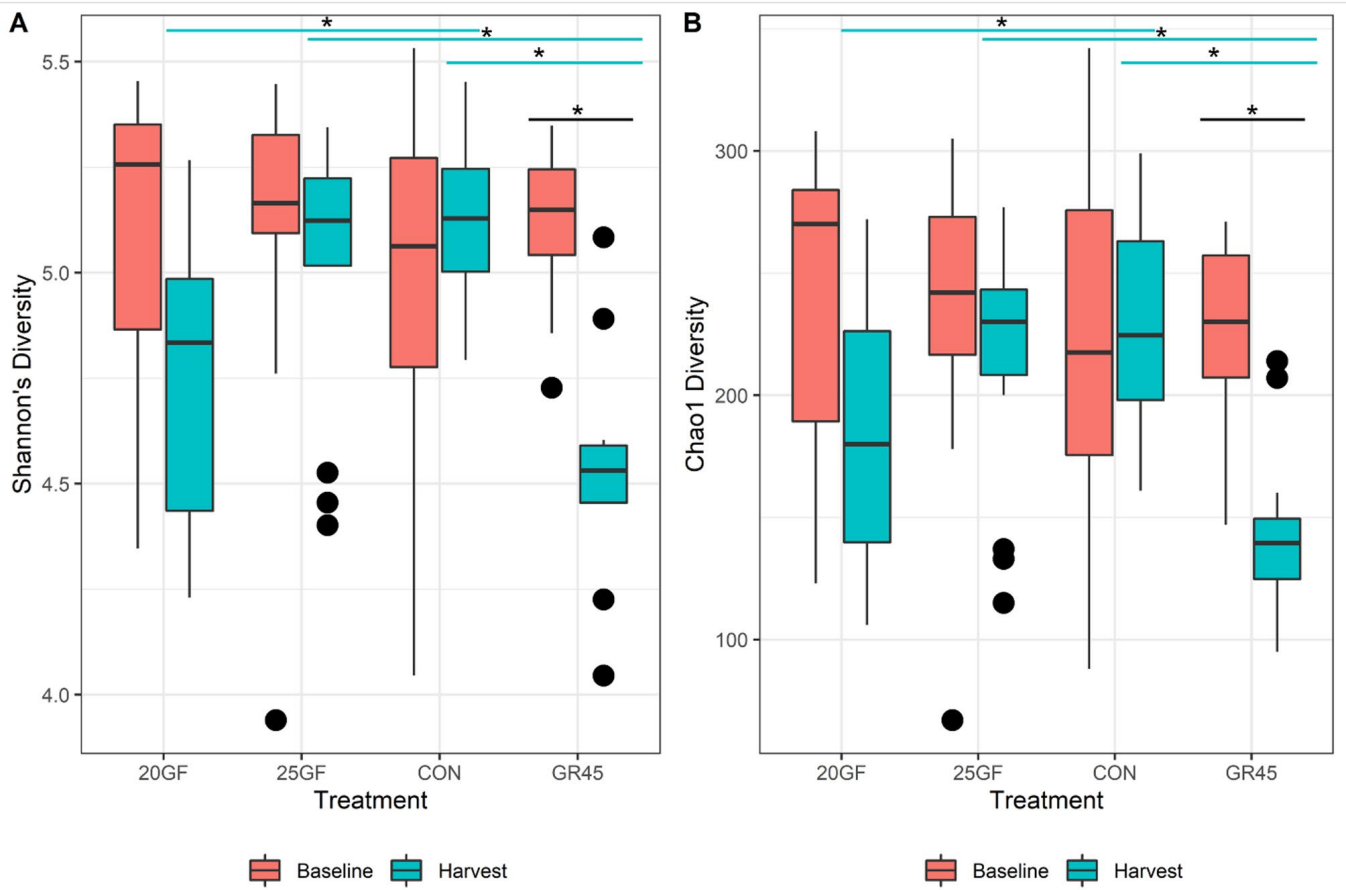
Beef cattle fecal alpha diversity was assessed by Shannon **(A)** and Chao1 **(B)** indices. Feeding system treatments: Conventional grain-fed (CON, *n* = 21), 20-months-grass-fed (20GF, *n* = 18), 20-months-grass-fed and 45-days-grain-fed (GR45, *n* = 13), 25-months-grass-fed (25GF, *n* = 16). * means significant (*P* < 0.05) difference between two groups by ANOVA followed by Tukey test for means separation * with a blue line shows the significant difference between treatments at harvest and * with a black line indicates the significant difference between baseline and harvest for GR45

While evaluating the fecal microbial alpha diversity between the feeding systems, similar results were obtained from both Chao1 and Shannon diversity index (Fig. 1). There were no differences (*P* > 0.05) in alpha diversity of fecal microbiome between feeding systems at baseline at the phyla level. At harvest, the CON steers feces had larger (*P* < 0.05) microbial alpha diversity values than the 20GF and GR45, whereas 25GF steers had larger (*P* < 0.05) alpha diversity of fecal microbiomes than GR45. The microbial alpha diversity of GR45 steers feces decreased (*P* < 0.05) from baseline to harvest, while CON, 25GF, and 20GF steers feces displayed no change (*P* > 0.05) from baseline to harvest. The fecal microbial alpha diversity remained the same for CON steers from baseline to harvest while the GR45 steers had lower (*P* < 0.05) alpha diversity of fecal microbiomes at harvest.

### Microbiome composition

**Fig. 2:**
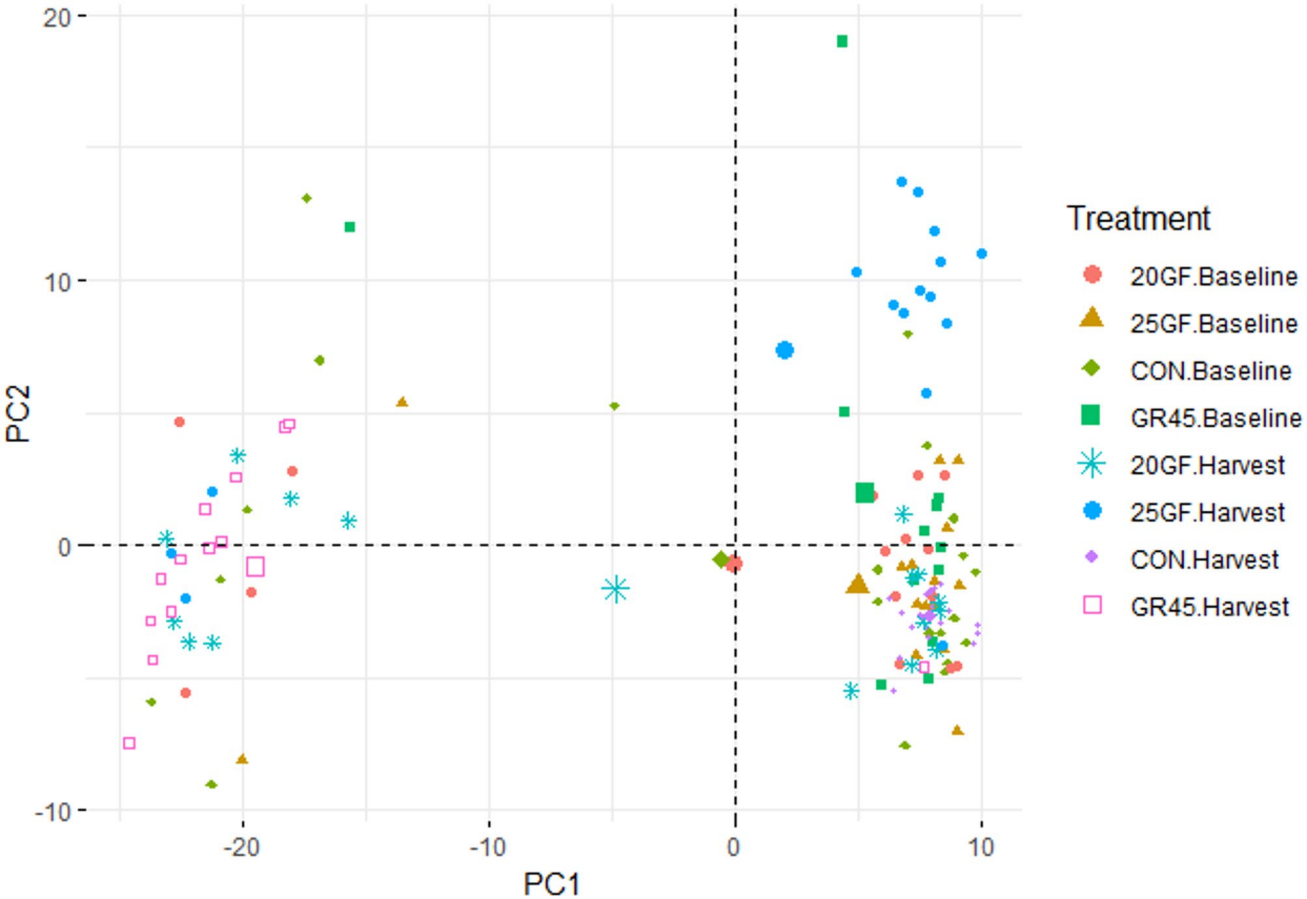
Principal Component Analysis of fecal microbiota of cattle raised under different feeding systems: Conventional grain-fed (CON, *n* = 21), 20-months-grass-fed (20GF, *n* = 18), 20-months-grass-fed and 45-days-grain-fed (GR45, *n* = 13), 25-months-grass-fed (25GF, *n* = 16)

At baseline, there was no difference in fecal microbial composition among the feeding systems (*R* = -0.022, *P* = 0.759), indicating that the cattle initially had similar fecal microbiome (Fig. 2).

**Fig. 3:**
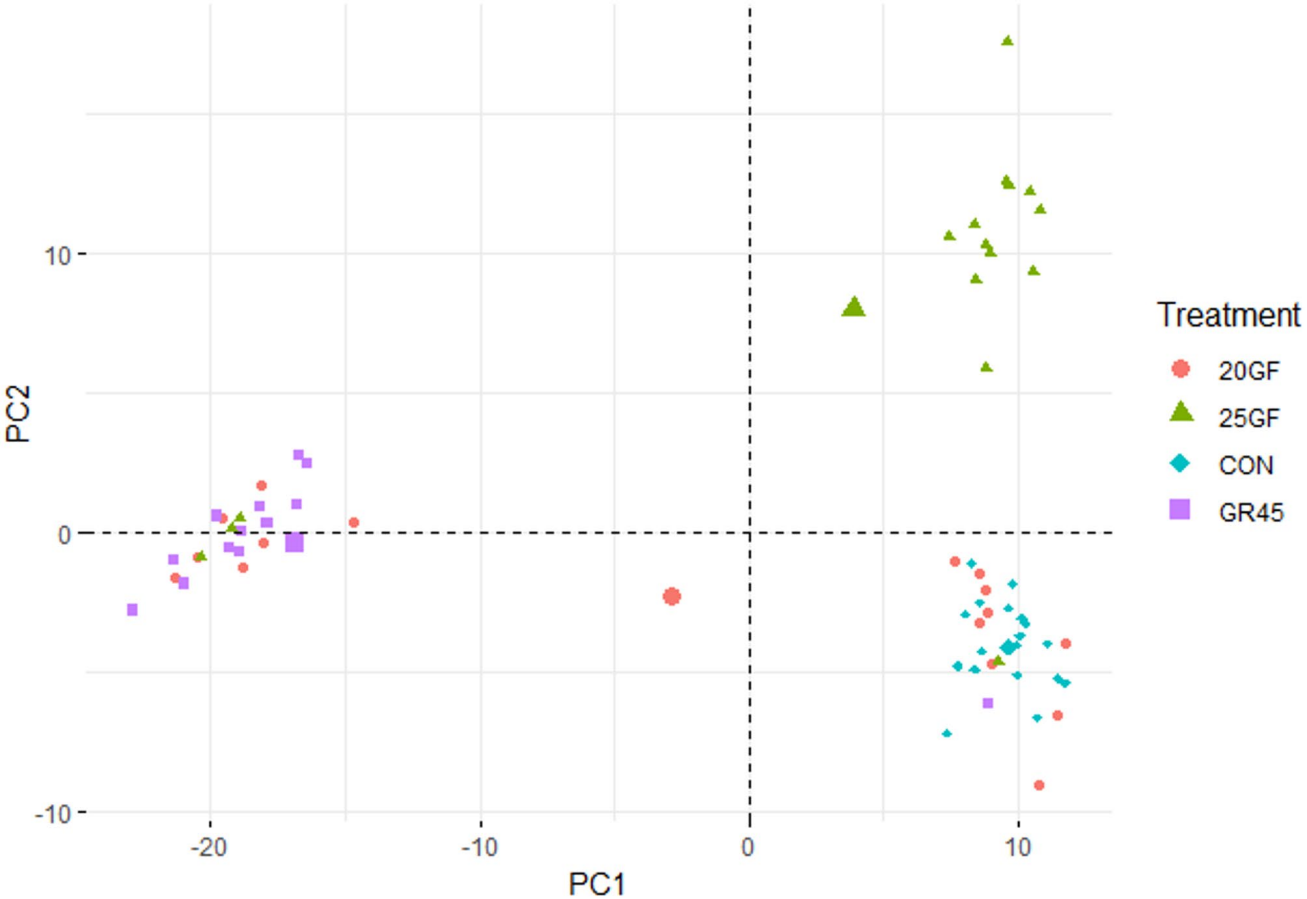
Principal Component Analysis of fecal microbiota at harvest of cattle raised under different feeding systems: Conventional grain-fed (CON, *n* = 21), 20-months-grass-fed (20GF, *n* = 18), 20-months-grass-fed and 45-days-grain-fed (GR45, *n* = 13), 25-months-grass-fed (25GF, *n* = 16)

At harvest, fecal microbial composition was revealed to be different among cattle raised in different feeding systems (*R* = 0.43, *P* = 0.001) (Fig. 3).

**Fig. 4:**
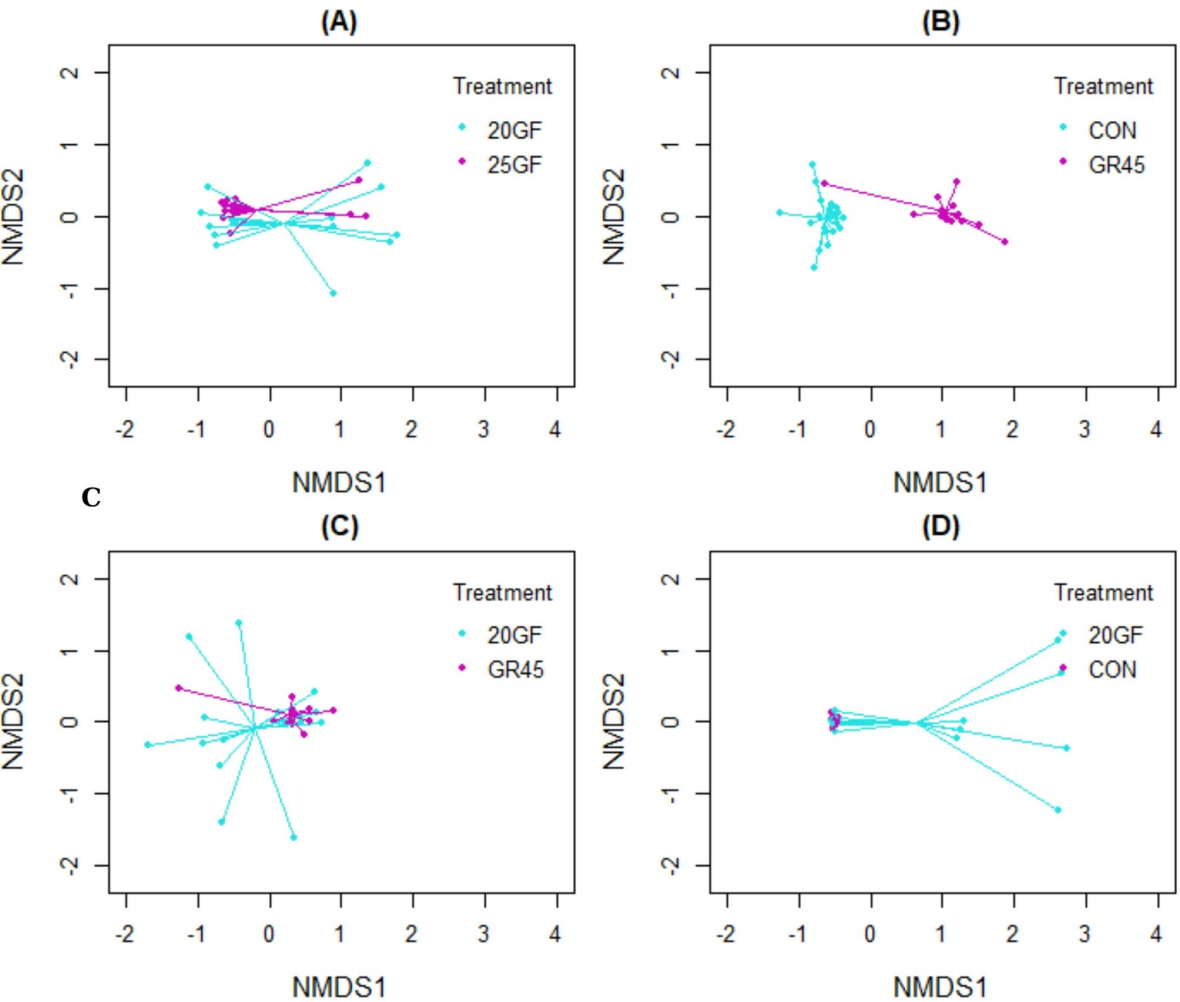
Non-metric multidimensional plot showing the fecal microbial composition of steers at harvest between **(A)** two grass-fed feeding systems, **(B)** CON and GR45, **(C)** 20GF and GR45, and **(D)** grass and conventional feeding systems. Feeding systems included: Conventional grain-fed (CON, *n* = 21), 20-months-grass-fed (20GF, *n* = 18), 20-months-grass-fed and 45-days-grain-fed (GR45, *n* = 13), 25-months-grass-fed (25GF, *n* = 16)

Comparison of the two grass-fed systems (20GF and 25GF) revealed overlap in fecal microbial composition at the time of harvest (*R* = 0.177, *P* = 0.013), as visually demonstrated in the NMDS plot in Fig. 4A. Additionally, it was surprising that the fecal microbial composition of GR45 was way similar to that of 20GF (*R* = 0.199, *P* = 0.013) than to CON (*R* = 0.913, *P* = 0.001), indicating that 45 days on conventional system didn’t quickly alter the microbiome of the adult cattle. The NMDS plot in Fig. 4B and C showed the distinct clustering of the samples between CON and GR45 steers while the separation was not obvious for the samples between 20GF and GR45. Comparison between two feeding systems (20GF vs. CON) at harvest showed some separation (*R* = 0.3001, *P* = 0.001) in their fecal microbial composition (Fig. 4D).

**Fig. 5:**
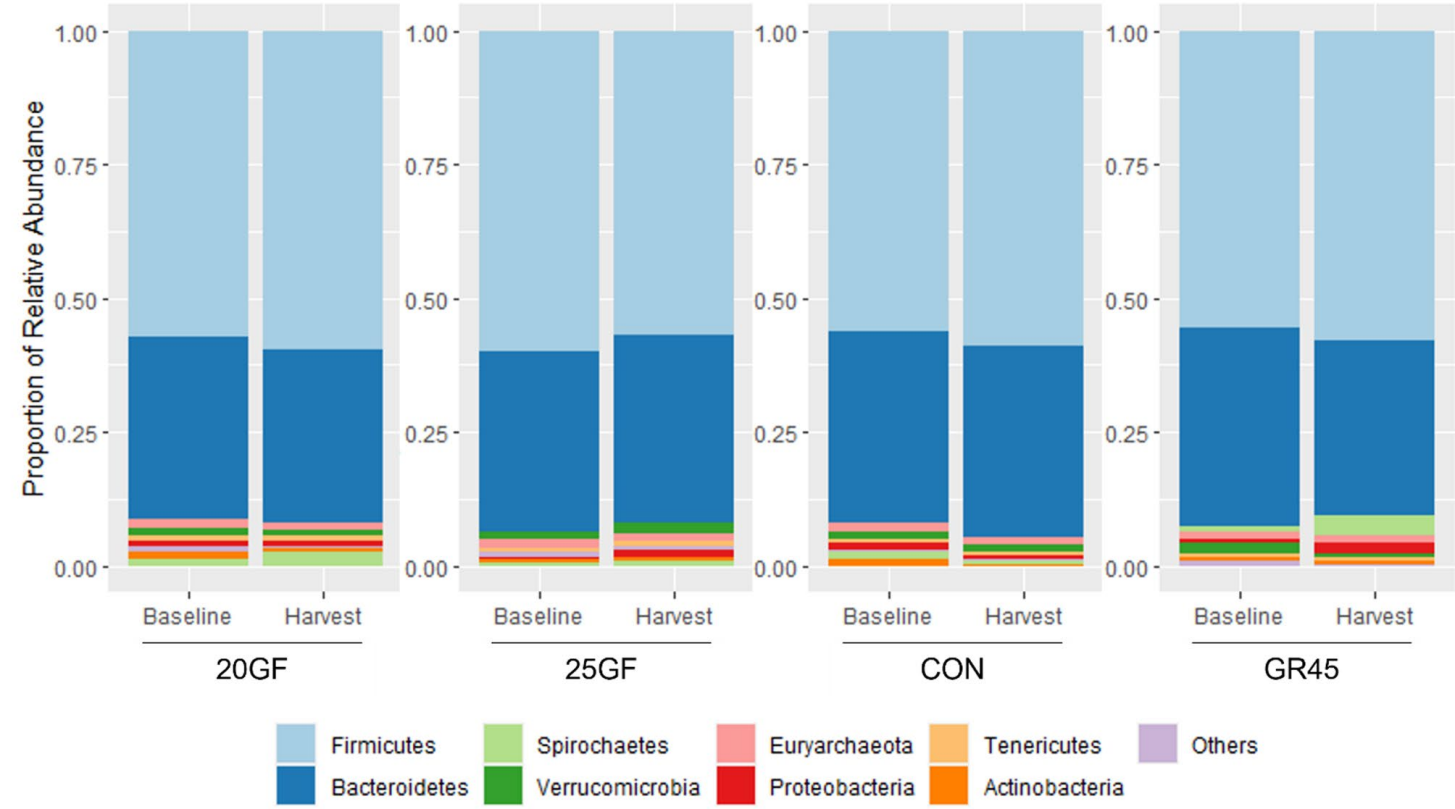
Stacked histograms indicating relative abundances of phyla in fecal microbiome of cattle raised under each feeding system at baseline and harvest. Feeding systems included: Conventional grain-fed (CON, *n* = 21), 20-months-grass-fed (20GF, *n* = 18), 20-months-grass-fed and 45-days-grain-fed (GR45, *n* = 13), 25-months-grass-fed (25GF, *n* = 16)). Eight primary phyla are shown in the figure. Phyla with a relative abundance of less than 0.50% are grouped as ‘Others’

The relative abundances of bacteria at the phyla level among samples at baseline and harvest are presented in Fig. 5. Bacteroidetes and Firmicutes were the most dominant phyla for each feeding system and sampling point, and the sum of the relative abundances of these two phyla captured more than 92% of sequencing reads. At baseline, the abundance of Firmicutes was lowest (*P* < 0.05) in the feces of cattle in GR45. However, at harvest, the abundance of Firmicutes was higher (*P* < 0.0001) in feces of cattle from GR45 followed by 20GF, compared to those from 25GF and CON.

**Fig. 6:**
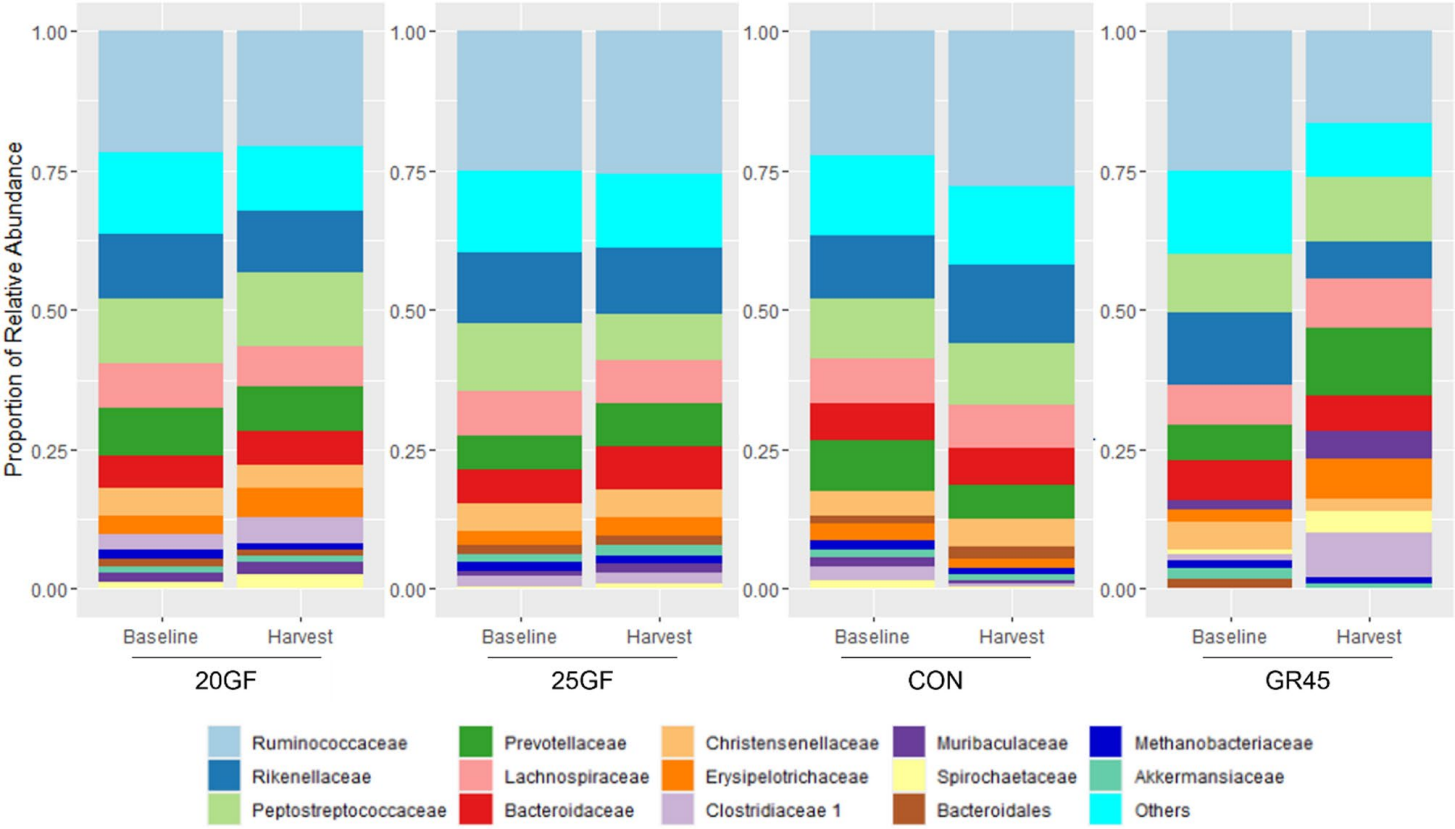
Stacked histograms indicating relative abundance of families in fecal microbiome of cattle raised under different feeding systems at baseline and harvest. Feeding system included: Conventional grain-fed (CON, *n* = 21), 20-months-grass-fed (20GF, *n* = 18), 20-months-grass-fed and 45-days-grain-fed (GR45, *n* = 13), 25-months-grass-fed (25GF, *n* = 16). 14 primary families are shown in the figure. Family with a relative abundance of less than 1% are grouped as ‘Others’

The relative abundances of several bacterial families (*Clostridiaceae*, *Erysipelotrichaceae*, *Muribaculaceae*, *Peptostreptococcaceae*, *Prevotellaceae*, *Rikenellaceae*, *Ruminococcaceae*, and *Spirochaetaceae*) shifted (*P* < 0.05) in feces of cattle raised in different feeding systems from baseline to the time of harvest (Fig. 6). For example, the relative abundance of *Prevotellaceae* decreased (*P* < 0.05), and the abundance of *Ruminococcaceae* increased (*P* < 0.05) in steers from CON at harvest. However, unlike other feeding systems, a substantial shift in bacterial families was observed for GR45 where the relative abundances of seven families (*Clostridiaceae*, *Erysipelotrichaceae*, *Lachnospiraceae*, *Muribaculaceae*, *Peptostreptococcaceae*, *Prevotellaceae*, and *Spirochaetaceae*) were increased (*P* < 0.05) over the lifespan, while the abundances of *Rikenellaceae* and *Ruminococcaceae* were decreased (*P* < 0.05). No change was observed in the relative abundances of bacteria at the family level in feces of the steers from 20GF.

**Fig. 7:**
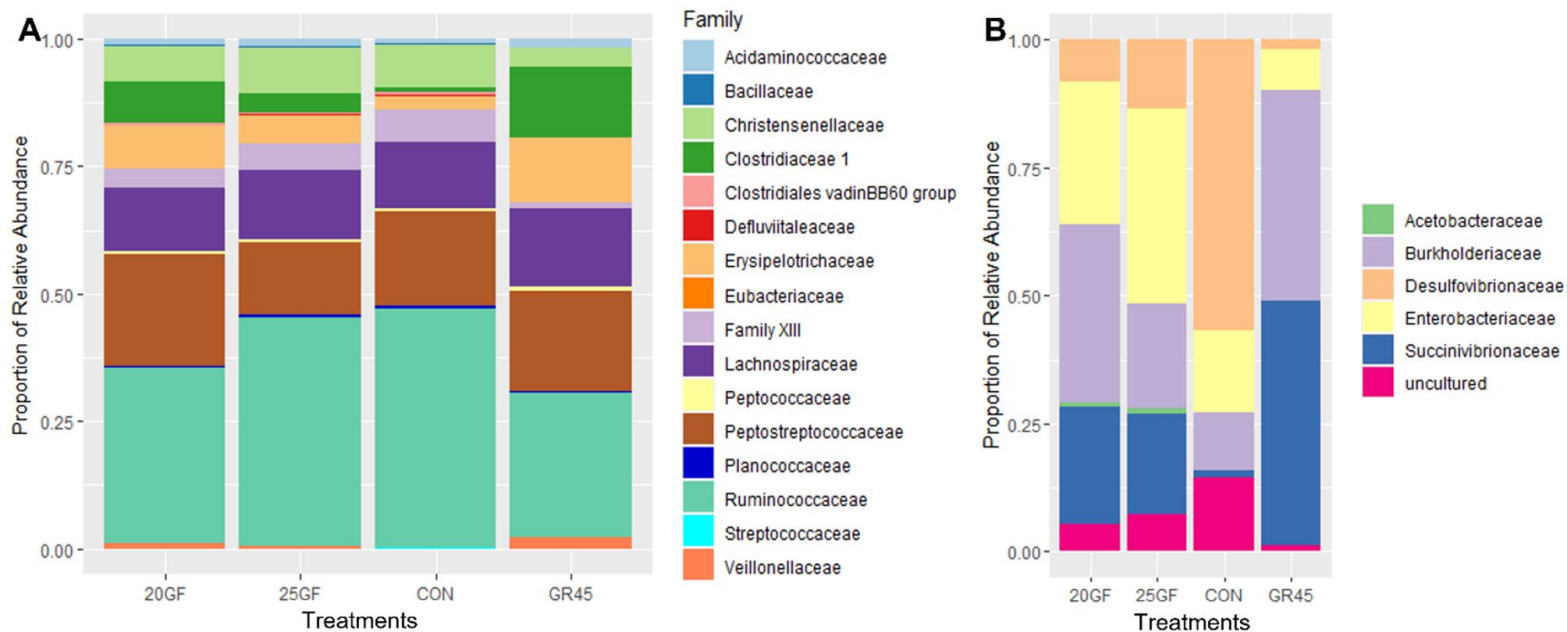
Stacked histograms indicating relative abundance of families of phyla **(A)** Firmicutes and **(B)** Proteobacteria in fecal microbiome of cattle raised under different feeding systems at harvest. Feeding systems included: Conventional grain-fed (CON, *n* = 21), 20-months-grass-fed (20GF, *n* = 18), 20-months-grass-fed and 45-days-grain-fed (GR45, *n* = 13), 25-months-grass-fed (25GF, *n* = 16)

Due to the significant prevalence of Firmicutes and the presence of Proteobacteria, which includes *E. coli*, a critical indicator bacteria for food safety, further analysis was conducted to better understand the role of these two phyla. The relative abundances of bacterial families of these two phyla are shown in Fig. 7. The *Clostridiaceae* abundance was the highest (*P* < 0.05) in the GR45 group, followed by 20GF, with both being higher (*P* < 0.001) than in the 25GF and CON groups. Steers from GR45 also had a higher (*P* < 0.05) abundance of *Erysipelotrichaceae* compared to the ones in 25GF and CON groups. *Peptostreptococcaceae* abundance was higher (*P* < 0.05) in 20GF compared to 25GF and CON, while *Ruminococcaceae* was more (*P* < 0.05) abundant in CON than in 20GF and GR45. For Proteobacteria, the abundance of *Burkholderiaceae* and *Succinivibrionaceae* families was highest (*P* < 0.05) in GR45, compared to all other feeding systems whereas *Enterobacteriaceae* abundance was higher (*P* < 0.05) in 25GF than in CON.

### Association between fecal microbiota and STEC population and prevalence

There was no (*P* > 0.05) correlation observed between the population or prevalence of STEC and the alpha diversity of the fecal microbiome. Similarly, no association (*P* > 0.05) was observed between the population or prevalence of STEC and the microbial composition (beta diversity) of the fecal microbiome analyzed from 16 S rRNA sequencing data. These findings suggest that the presence or the abundance of STEC was not associated with the overall microbial diversity metrics in the samples analyzed. However, when comparing the difference in relative abundance of bacterial phyla based on the presence of STEC, only Bacteroidetes showed (*P* < 0.05) difference. Specifically, STEC-negative samples exhibited higher (*P* < 0.05) abundance of Bacteroidetes. Within this phylum, the bacterial families *Prevotellaceae* and *Muribaculaceae* were found to be higher (*P* < 0.05) in STEC-negative samples compared to STEC-positive samples.

## Discussion

The increasing preference of consumers towards grass-fed beef over grain-fed beef emphasizes the significance of comprehending the effects of these feeding systems on the microbial communities in cattle gut and the prevalence of foodborne pathogens. This study aimed to comprehensively evaluate the effects of grain-fed and grass-fed feeding systems on the prevalence and population levels of STEC, as well as the composition and diversity of the fecal microbial communities in beef cattle. In the current study, the grass-fed feeding system was observed to be associated with a lower fecal population of STEC in steers compared to the grain-fed feeding system. This finding aligns with previous studies that concluded cattle finished on grass diets exhibited lower gut and fecal STEC populations, as indicated by the naturally occurring *E. coli* population [39]. Nevertheless, prior investigations have yielded inconsistent results when comparing the effects of grain and grass-fed feeding systems on fecal STEC populations [18, 19, 39–41]. These contrasting findings reflect the lack of a clear consensus in the literature regarding the impact of forage- versus grain-based diets on STEC shedding in cattle. These discrepancies may also reflect differences in study design, diet composition, cattle age, environmental conditions, or sample timing, highlighting the complexity of interpreting nutrition-pathogen interactions in cattle.

The observed higher fecal STEC population in CON and GR45 steers in the present study might have been influenced by the inclusion of monensin and distillers’ grain (DG) in the grain-feeding regimens, which are common components of conventional feeding systems. Contrasting these two feeding systems, the 20GF and 25GF steers were grass-fed, monensin or DG was not incorporated into their respective diets. Monensin, an ionophore, is frequently administered to cattle in confined feeding environments as an antimicrobial compound [42]. It has been shown that monensin has the ability to inhibit the growth of gram-positive bacteria, which has been suggested to confer a competitive advantage to gram-negative bacteria such as *E. coli* [42]. However, the majority of studies did not observe a correlation between monensin feeding and fecal STEC levels in cattle on corn-based rations when added for a duration between 2 and 11 weeks, which may have been insufficient to induce measurable microbial changes affecting STEC shedding [19, 43, 44]. The present study was modeled as real-life settings where monensin supplementation was used in grain-fed CON steers when they were raised on the feedlot for 17 to 18 weeks. This longer duration of using monensin supplementation may account for the difference in results, as a longer duration of added monensin of 18 weeks has previously been shown to have greater enumerable fecal STEC population than the control [45]. Long-term monensin supplementation could modify the gut environment, potentially changing factors like pH or short-chain fatty acid concentrations, which might indirectly benefit STEC growth [46]. The inclusion of DG in cattle diets has been examined for its potential effects on STEC dynamics, with some studies suggesting prolonged survival of *E. coli* in manure [47], while others have reported no significant impact on fecal shedding in live animals [48]. In the present study, DG was included in the conventional diet fed to both CON and GR45 steers. Notably, these two groups also exhibited higher fecal STEC populations compared to grass-fed steers, though the extent to which DG contributed to this observation remains unclear. In addition to these dietary components, it is important to note that grass-fed cattle in this study were older at harvest than their grain-fed counterparts. Previous research suggests that STEC shedding tends to decline with age, potentially due to immune maturation or stabilization of the gut microbiota [49]. Therefore, differences in STEC population between feeding systems may also have been influenced by age-related effects, which should be considered when interpreting the results.

Earlier propositions have indicated that decreased pH and elevated concentrations of volatile fatty acids (VFA) do not impact the survival of STEC in the rumen [50], but these conditions may favor the survival of acid-resistant STEC strains [51]. Consequently, one might anticipate an increased prevalence of acid-resistant STEC in cattle on a grain-fed regimen. However, in the present study, the acid-resistant STEC prevalence in CON steers decreased to zero, while the overall STEC prevalence was not changed (*P* > 0.05) as the steers grew. A previous study concluded that the development of acid resistance of STEC is unrelated to the low acidity in the rumen, suggesting that the transformation from acid-sensitive to acid-resistant can occur after passing through the rumen and is not diet-dependent [52]. Also, the samples collected for this study were from the rectum, not rumen, which might also be a substantial fact considering that the STEC shedding can be varied in different regions of the gastrointestinal tract. The STEC population and prevalence observed in the rectal samples may not necessarily reflect the overall STEC status throughout the entire gut of the steers. It is important to note that while the rectal samples may not represent the entire gastrointestinal tract, they hold particular significance in the context of food safety. The feces excreted from the rectum are the primary source of environmental contamination and pose the greatest risk for food contamination during slaughter and processing. Therefore, the STEC population and prevalence observed in rectal samples are crucial indicators of the potential for contamination in the food supply chain. The observation of 100% STEC prevalence at baseline across all treatment groups is notably high and not commonly reported in naturally colonized cattle. Co-housing of all the steers before the initiation of the trial may have facilitated horizontal transmission of STEC among individuals. Additionally, environmental exposure from shared pasture or water sources prior to the feeding trial may have contributed to widespread colonization. While the cattle were managed according to typical grass-fed practices in California, no testing of irrigation or drinking water was performed during this period, limiting our ability to evaluate waterborne exposure as a contributing factor.

When comparing the alpha diversity of fecal microbiomes between different feeding systems, the observed effect does not appear to be inherently linked to the conventional or grass-fed feeding systems. Most of the previous studies have reported more diverse fecal bacterial communities in grass-fed cattle compared to grain-fed cattle [22, 23]. However, in the present study, at harvest, steers from CON had a higher (*P* < 0.05) alpha diversity than the 20GF and GR45 group, followed by 25GF which had a higher (*P* < 0.05) alpha diversity than GR45. The disparity between the result to previous reports could be partly attributed to the seasonal effects and higher temperatures during fecal sample collection, particularly during the summer months in California, as heat stress during this period has been associated with reduced microbial diversity in cattle [53]. In the current study, fecal samples collected from CON steers in April and from 25GF steers in October occurred at relatively lower average daily temperatures which might be attributed to the observed higher alpha diversity in the steers from these two feeding systems. From 20GF and GR45 steers, fecal samples were collected in June and August when there was a historic heat wave reported in the southwest part of California which resulted in around 10ºF higher average daily temperature than in April or October in the Sacramento area [54]. In addition to environmental temperature, differences in pasture composition between studies may also contribute to the variation in microbial diversity. The grass-fed steers in our study grazed on seasonal California pastures that likely differed in species composition and nutritional profile from those used in previous studies [22, 23]. Variation in fiber content, protein levels, and secondary metabolites across forage types can influence gut microbial structure and may help explain discrepancies in alpha diversity trends. Moreover, the significant dietary shift in the GR45 feeding system may also contribute to this change (*P* < 0.05) in fecal microbial diversity from baseline to harvest.

The beta diversity of fecal microbiomes at harvest indicated compositional dissimilarity among the feeding systems. Most of the published research primarily focused on the comparison between the fecal microbiome of grain and grass-fed cattle where distinct microbial diversity was observed in the feces of grass-fed cattle compared to grain-fed cattle [22, 23, 55]. Aligned with the reported studies, our findings also revealed a separation (CON vs. 20GF: *R* = 0.3001, *P* = 0.001; CON vs. 25GF: *R* = 0.474, *P* = 0.001) in microbial composition between the conventional feeding system and grass-fed feeding systems at harvest. Interestingly, distinct fecal microbial compositions were observed when comparing the conventional feeding system and GR45 steers (*R* = 0.913, *P* = 0.001) at harvest. However, similar microbial composition was observed in 20GF and GR45 (*R* = 0.199, *P* = 0.013). This relatively similar microbial composition between 20GF and GR45 steers raises an important question about the time required for significant microbiome shifts. While 45 days of conventional feeding did induce some changes, it may not have been sufficient for a complete transformation of the microbiome. Previous studies have shown that diet-induced shifts in the rumen microbiome begin within four weeks and take almost nine weeks for microbiome stability, but the rate and extent of change can vary depending on the specific dietary components and individual animal factors [56]. The fecal microbiome, which reflects both ruminal and hindgut populations, may require a longer adaptation period. Additionally, the prior long-term grass feeding in GR45 steers might have established a more resilient microbial community, potentially slowing the transition to a grain-associated microbiome.

In the present study, no significant correlation was observed between the population or prevalence of STEC and the alpha or beta diversity of the fecal microbiome. This suggests that the overall microbial diversity may not be directly linked to STEC presence or abundance in cattle feces. However, when examining specific bacterial groups, we found that STEC-positive samples had a significantly higher abundance of Bacteroidetes, particularly the families *Prevotellaceae* and *Muribaculaceae*. This contrasts with some previous studies that reported increased bacterial diversity associated with decreased STEC shedding [24, 25]. Our findings indicate that while overall diversity may not be a determining factor, specific bacterial families within the Bacteroidetes phylum might play a role in STEC colonization or persistence in the bovine gut. At phyla level, the dominant phyla found were Firmicutes and Bacteroidetes. Clostridiaceae, a dominant family found in the bovine gut microbiome, has been observed to be associated with STEC shedding in cattle. Specifically, studies have found that STEC shedders have a higher proportion of members from the order Clostridiales [24, 57]. However, no difference in its abundance was found based on STEC prevalence in our study. Other phyla such as Bacteroidetes and Proteobacteria are more prevalent in the gut of grass-fed cattle because of their function to break down fiber [58]. Nevertheless, a previous study also revealed a higher abundance of Bacteroidetes in the feces of grain-fed cattle than in grass-fed cattle [59]. However, no significant difference in Bacteroidetes and Proteobacteria abundance was found at harvest among different feeding systems in our study. This unlikely outcome may be due to the variable relative abundance of Bacteroidetes at the baseline along with other factors such as preference for certain substrates in diet, differences in grass types, animals’ genetics, and the management practices used [60]. It is also important to note that in the present study, samples were collected from the rectum, which may not necessarily reflect their functional extent in the rumen. A higher abundance of Bacteroidetes was found in STEC-negative samples in this study. In a previous study conducted on humans infected with acute enteric infections caused by STEC, the abundance of Bacteroidetes was found to be lower compared to healthy communities whereas the abundance of Proteobacteria was higher in STEC-infected stool samples [61]. While these findings provide important insight into how feeding systems shape fecal microbial communities, it is important to acknowledge that rectal samples may not fully capture the microbial diversity and functional potential of the entire gastrointestinal tract. The gut microbiota differs substantially across regions such as the rumen, small intestine, and colon due to variations in pH, oxygen availability, and nutrient gradients. Therefore, conclusions drawn from rectal microbiota may underrepresent microbial diversity and function in more proximal gut sections.

It’s crucial to acknowledge the limitations of the 16 S rRNA sequencing method used in this study. The relatively low resolution of this technique may have prevented us from detecting specific microbiome associations with STEC-positive or negative samples at lower taxonomic levels. The 16 S rRNA sequencing typically provides reliable classification at the phylum and family levels but may lack resolution for genus or species-level identification [62].Additionally, CHROMagar STEC, while widely used for isolating *eae*-positive STEC, including many clinically relevant serogroups, has limited sensitivity for detecting *eae*-negative strains [63]. As such, the STEC populations captured in this study are most representative of *eae*-positive variants. It is also important to note that many previous studies have specifically focused on *E. coli* O157:H7, whereas our study targeted STEC as a broader group based on the presence of *eae* and either of the *stx* genes. Given the phenotypic diversity among STEC serogroups, direct comparisons across studies should be interpreted with caution. Future studies incorporating broader isolation methods and serogroup-specific resolution may help better characterize the full spectrum of STEC in cattle.

It is also important to consider that STEC represents only a small subset of the generic *E. coli* population. As a result, the relative presence of STEC within generic *E. coli* populations is an important distinction when interpreting fecal microbial community dynamics, even at the species or strain level. Overall, the differences observed in microbial composition among feeding systems and in relation to STEC status in our fecal samples underscore the complex interactions within the bovine gastrointestinal tract. While these findings provide valuable insights, they also highlight the need for further research using more advanced sequencing techniques to fully elucidate the relationship between feeding systems, fecal microbiota, and STEC prevalence in cattle.

## Conclusions

This study contributes to a growing body of evidence showing that beef cattle feeding systems influence fecal STEC dynamics and gut microbial composition, both of which are critical to understanding and mitigating foodborne pathogen risks. While grass-fed cattle exhibited lower fecal STEC populations, there was no definitive evidence to support the superiority of this system in reducing overall STEC or acid-resistant STEC prevalence. Additionally, the use of monensin and distillers grains in conventional feeding systems may significantly influence STEC shedding, indicating the need for further investigation. The observed reduction in STEC prevalence in the grain-finished (GR45) group suggests that short-term dietary modifications could offer a practical pre-harvest intervention strategy. These insights may inform feedlot management decisions and support regulatory efforts to reduce STEC contamination at slaughter. Future research should explore how longer grain-finishing durations or different forage-to-concentrate transitions influence both STEC shedding and gut microbiota across different regions of the gastrointestinal tract. Additionally, integrating metagenomics or metabolomics could offer deeper insights into the functional roles of microbial communities in pathogen suppression. Overall, the distinct bacterial compositions in cattle raised under different grass-fed and grain-fed feeding systems do not necessarily negate the influence of other factors, such as animal management practices, animal age, season, and expected external stresses, which can interfere with the fecal microbial population. Thus, to accurately evaluate the risk of foodborne pathogens transmission through animal fecal materials, it is essential to take into account a comprehensive assessment of all relevant factors that impact the safety of end products.

## Data Availability

The datasets generated during this study are available in the National Center for Biotechnology Information (NCBI) database under accession numbers MZ521235 - MZ522111.
